# Parkinson's disease is characterized by sub-second resting-state spatio-oscillatory patterns: A contribution from deep convolutional neural network

**DOI:** 10.1016/j.nicl.2022.103266

**Published:** 2022-11-13

**Authors:** Mehran Shabanpour, Neda Kaboodvand, Behzad Iravani

**Affiliations:** a Zanjan University of Medical Sciences, Zanjan, Iran; b Department of Clinical Neuroscience, Karolinska Institutet, Stockholm, Sweden; c Department of Neurology and Neurological Science, Stanford University, Stanford, United States

**Keywords:** Convolutional neural network, Resting-state oscillation, Parkinson’s disease, Motor cortical beta activity, Frontoparietal theta power

## Abstract

Deep convolutional neural network (DCNN) provides a multivariate framework to detect relevant spatio-oscillatory patterns in the data beyond common mass-univariate statistics. Yet, its practical application is limited due to the low interpretability of the results beyond accuracy. We opted to use DCNN with a minimalistic architecture design and large penalized terms to yield a generalizable and clinically relevant network model. Our network was trained based on the scalp topology of the electroencephalography (EEG) from an open access dataset, constituting our primary sample of healthy controls (n = 25) and Parkinson’s disease (PD) patients (n = 25), with and without medication. Next, we validated the model on another independent, yet comparable open access EEG dataset (healthy controls (n = 20) and PD patients (n = 20)), which was unseen to the network. We applied Gradient-weighted Class Activation Mapping (Grad-CAM) interpretability technique to create a localization map exhibiting the key network predictors, based on the gradients of the classification score flowing into the last convolutional layer. Accordingly, our results indicated that a sub-second of intrinsic oscillatory power pattern in the beta band over the occipitoparietal, gamma band over the left motor cortex as well as theta band over the frontoparietal cluster, had the largest impact on the network score for dissociating the PD patients from age- and gender-matched healthy controls, across the two datasets. We further found that the off-medication motor symptoms were related to the occipitoparietal off-medication beta power whereas the disease duration was associated with the off-medication beta power of the motor cortex. The on-medication theta power of the frontoparietal was related to the improvement of the motor symptoms. In conclusion, our method enabled us to characterize PD patho-electrophysiology according to the multivariate topographic analysis approach, where both spatial and frequency aspects of the oscillations were simultaneously considered. Moreover, our approach was free from common reference problem of the EEG data analyses.

## Highlights

• Sub-second resting state EEG is impaired in Parkinson’s disease (PD).
• Occipitoparietal beta pattern dissociates the healthy controls from PD.
• Gamma pattern over the motor cortex dissociates PD off medication.
• Occipitoparietal and motor beta power are associated with the PD characteristic.
• On-medication frontoparietal theta power predicts improvement in motor symptoms.

## 1 Introduction

Understanding the pathophysiology of Parkinson’s disease (PD) is crucial for improving the diagnosis and treatment. Numerous studies have attempted to identify the abnormal signatures of brain dynamics related to the pathophysiology of PD, yet several controversial findings have been reported. PD is often characterized by a slowing of brain oscillations involving theta, beta and gamma frequency bands (Bosboom et al., 2006, Stoffers et al., 2007, Vardy et al., 2011, Vinding et al., 2020), although some other studies point at the reverse direction (Crowell et al., 2012, O’Keeffe et al., 2020, Pollok et al., 2012). Similarly, there are inconsistent findings regarding the effect of treatments in PD, so that there are reports of both increased cortical beta power following the administration of dopaminergic medication (Heinrichs-Graham et al., 2014, Melgari et al., 2014) or deep brain stimulation (Airaksinen et al., 2012, Cao et al., 2017), and a broad suppression of 5–25 Hz power in frontal and sensorimotor cortex in patients treated by deep brain stimulation (Abbasi et al., 2018, Luoma et al., 2018).

There are notions from both theoretical and empirical aspects to support that the ongoing spontaneous brain activity has well-structured spatiotemporal dynamics which is imperative to understand the brain function in health and disease (Fox and Raichle, 2007, Fransson, 2005, Kaboodvand et al., 2020, Smith et al., 2009). Nevertheless, most of the existing resting-state research is conceived from functional magnetic resonance imaging (fMRI). Overrepresentation of fMRI in the literature not only limits our quantitative understanding of the brain dynamics due to its low temporal resolution, but also impedes applicability of any derived biomarker for closed-loop neuromodulation due to the requirement of several minutes of imaging to achieve reliable estimations of brain network characteristics (Birn et al., 2013). There has been a growing momentum to use electroencephalography (EEG) data, although EEG comes with its own limitations.

One of the major drawbacks of EEG is the reference problem (Hari, 2010). Accordingly, the potential fluctuations of the reference electrode are reflected into the active electrodes and therefore their potential is dependent on the choice of reference. However, the pattern of the EEG topographic maps is reference independent (Geselowitz, 1998), and therefore the disproportionate stress on the analysis of EEG waveforms regardless of the topology of the scalp potentials has been hampering the analytic power of EEG (Michel and Murray, 2012). This highlights the importance of further developing multivariate analysis methods, for characterizing the topology of resting EEG (rs-EEG) potentials.

In previous years, efforts have been devoted to improving multivariate analysis methods using machine learning (ML) algorithms. Among various ML approaches, deep convolutional neural networks (DCNNs) are well capable of pattern recognition in highly multivariate data (Dhillon and Verma, 2019, LeCun et al., 2015). It has been further indicated that DCNNs outperform the other conventional ML approaches such as naïve Bayes or support vector machine (Dhillon and Verma, 2019). Yet, they are underused in the neuroscience field, mainly due to the lack of large datasets and also unintuitive feature maps of the convolutional layers that make the interpretation of the results beyond the classification accuracy nearly impossible. Nevertheless, DCNNs have been used, for example, for the recognition of the emotional state of PD patients (Dar et al., 2022) or to classify PD from the healthy individuals (Lee et al., 2021, Oh et al., 2018). The scope of ML research for PD has been usually limited to the common “black box” perspective with a large parameter space (i.e., tens to hundreds of thousands) and have not been providing any further insight into the pathology of the disease.

Contrary to most of the previous ML research on PD, here we went beyond the classification of our sample to PD and healthy, to shed light on the electrophysiological signature that is involved in PD, using the multivariate nature of DCNNs. Critically, we were mindful of the pitfalls of using DCNNs on datasets with a limited number of individuals. We hypothesized, by taking certain steps that will be discussed under the network generalizability section, a three-dimensional DCNN (3D-DCNN) with a minimum possible number of trainable parameters could robustly characterize PD based on brief 1-second-long rs-EEG segments. It has been recently shown that the convolutional networks are capable to extract enough information from only 1-second-long EEG data to, for example, predict the recognition of emotions in PD cohort (Dar et al., 2022). Nevertheless, given the natural susceptibility of the EEG to various artifacts, obtaining a high classification accuracy is contingent to the overfitting problem. We favoured generalizability of our results rather than obtaining a high accuracy by taking a minimalistic approach in our network design. We further assessed the generalizability of our findings by replicating them on an independent data, novel to our network. Finally, we applied Gradient-weighted Class Activation Mapping (Grad-CAM) to interpret the deep learning model (Selvaraju et al., 2019) trained to classify the healthy control and PD, providing a viable roadmap to the clinical translation of this methodology. This interpretability technique explains the network predictors by using the gradient information flowing into the final convolutional layer and returns a score map that outlines parts of the input data that has the most impact on classification.

## 2 Materials and methods

### 2.1 Primary dataset and pre-processing

We used an open access EEG dataset (OpenNeuro Dataset ds003490), available on the OpenNeuro platform, provided by Cavanagh and colleagues (Cavanagh, 2021). The data was collected around 2015 in Cognitive Rhythms and Computation Laboratory at University of New Mexico. EEG data from a total of 50 individuals, including PD patients (n = 25), as well as age- and gender-matched controls (n = 25, so-called Heathy control), were recorded from 64 channels. The patient cohort accomplished two sessions that were a week apart, with either off or on medication. Correspondingly, from this point forward, the PD cohort off-medication is simply referred to as PD OFF MED and the PD patients on-medication are referred to as PD ON MED. The PD OFF MEDs were appointed to withdraw their prescribed dopaminergic medication for 15 hours prior to the data collection session. It is worth noting that the Unified Parkinson's Disease Rating Scale (UPDRS) of PD patients have been assessed by a neurologist (Cavanagh et al., 2018). Moreover, all participants accomplished Mini Mental State Exam (MMSE), indicating no individual scoring below 26. Critically, the two cohorts did not differ in MMSE on the group level (i.e., MMSE PD = 28.68 ± 1.03, MMSE Healthy control = 28.76 ± 1.05, *t*(48) = 0.27, *p* >.79, *CI* = [-0.67, 0.51]). Table 1 presents a demographic overview and summarizes the clinical characteristics of the PD cohort in the primary dataset.

**Table 1:** The demographic overview of the PD and Healthy control participants, as well as a summary of the PD clinical data in the primary dataset (mean ± standard deviation).

	PD	Healthy control
N	25	25
Age	69.98 ± 8.73	69.32 ± 9.58
Gender	16 men, 9 women	16 men, 9 women
Disease duration	5.40 ± 4.09 (years)	–
LEDD^a^	685 ± 452 (mg)	–
UPDRS^b^ motor, on	Median: 22, range: [5, 40]	–
UPDRS motor, off	Median: 24, range: [10, 41]	–

a LEDD: levodopa equivalent daily dose.

b UPDRS: unified Parkinson’s disease rating scale.

All participants included in this study had completed EEG recording sessions comprised of 2 min of rs-EEG, with 1-minute-long eyes-open and eyes-closed conditions (i.e., 50% of trials were allocated to each of these rest conditions), and 3-stimulus auditory oddball task (Cavanagh et al., 2018). Here, we only focused on the resting-state data. The assessment of the task data has been summarized elsewhere (Cavanagh et al., 2018).

**Fig. 1:**
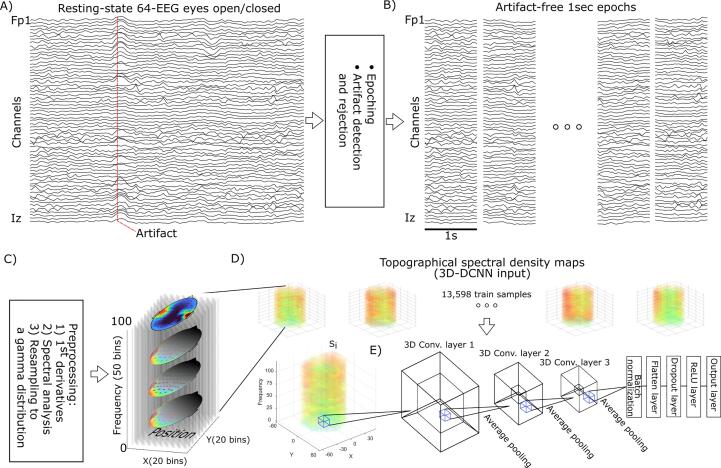
**Method summary. A**) The continuous resting-state eyes-open/-closed EEG (rs-EEG) were examined for artifacts and the segments of data with artifacts were flagged. **B**) The continuous rs-EEG was segmented into 1-second-long trials and the ones containing artifacts were discarded. **C**) Topographic spectral maps were derived from the first temporal derivatives of the rs-EEG and then normalized to a gamma distribution. **D**) One topographic map per frequency bin was achieved, followed by stacking the matrices into the tensors serving as the input volume of the 3D-DCNN. **E**) Each convolutional layer in the 3D-DCNN architecture is represented by a tesseract. The output of the last convolutional layer was batch normalized and flattened prior to applying the dropout with the rate of 50%. After passing through the dropout layer, the resulting sparse output was fed into a Rectified Linear Unit (ReLU) layer followed by a softmax output layer with 3 neurons.

In order to increase the generalizability of our findings, we applied only necessary pre-processing steps including mean-centring, removing power line noise (i.e., 60 Hz), re-referencing to the average of electrodes and computing the first temporal derivatives to control for 1/f noise. Moreover, we included both eyes-open and eyes-closed conditions to further remove the dependency of our findings on the particular states of the resting-state and the drifts in the visual attention (Boytsova and Danko, 2010). Next, we visually assessed the 64-channel EEG data and flagged the trials with a large amplitude as artifacts (Fig. 1A). This step concerned the blink artifacts that were clearly visible in the raw EEG data. We opted not to use automatic methods such as independent component analysis, to comply with our approach to keep the pre-processed data as close as possible to the raw data. We subsequently epoched the data into 1-second-long trials and therefore, we achieved 120 trials per individual. We further excluded trials that included our previously identified artifacts (Fig. 1B). On average, we kept 102 ± 15, 105 ± 20 and 100 ± 8 artifact-free trials, respectively for Healthy control, PD OFF MED and PD ON MED. Notably, the number of artifact-free trials did not statistically differ across three cohorts, as determined by one-way ANOVA, *F*(2, 72) = 0.6, *p* = 0.55. Moreover, the noisy electrodes were visually detected and interpolated using the nearest neighbouring electrodes. We tried to limit the number of interpolated electrodes by choosing a liberal threshold. On average, we interpolated 3 ± 3 electrodes per individual. Next, the first temporal derivative of EEG was calculated to diminish the non-oscillatory 1/f spectral background (Menceloglu et al., 2021, Menceloglu et al., 2020, Niederhauser et al., 2003). Considering that the data is collected during the resting-state and no clear baseline can be defined to remove 1/f noise, we used the first temporal derivative of EEG as an alternative solution to the baseline normalization. The pre-processed rs-EEG epochs were de-trended and a single hanning window was used to estimate the power in the frequency range of 1–100 Hz with the step-size of 2 Hz. Moreover, the spectral density (i.e., the power values) were resampled to a standard gamma distribution (*Γ(1, 0.5)*) to ensure that absolute values were in a unified range while keeping the general spectral pattern of the data (Fig. 1C). The EEG pre-processing was performed using the open source FieldTrip toolbox (Oostenveld et al., 2011).

### 2.2 Topographic spectral analysis

The topographic spectral maps were generated by mapping the spectral density of each channel at each frequency bin to the corresponding scalp position derived from the standard electrode positions in the 10–20 EEG layout. We further used the nearest neighbourhood 2D interpolation to transform the scattered sampled data into a continuous form and generated the 20 × 20 topographic maps (Fig. 1D). Not to mention that the interpolation of the data to the 2D plane was necessary to be able to define a neighbourhood of the channels and to encode the spatial information in a clinically meaningful manner. Hence, a total of 50 topographic maps, one per frequency bin, were defined per trial. We further stacked the topographic maps into the 3D volumes, where the transverse plane represented the positions on the scalp and the z-axis represented frequencies (Fig. 1D). These 3D volumes constituted the inputs to our 3D-DCNN.

### 2.3 3D deep convolutional neural network architecture

We sought to use the 3D-DCNN to characterize the main effects of PD and medication on rs-EEG measurements. Accordingly, we used a DCNN with three 3D-convolutional layers and a “sigmoid” activation function (Fig. 1E). The input volumes had dimensions of 20 × 20 × 50 (i.e., position(x) × position(y) × frequency). However, the convolutional layers allowed us to keep the size of the feature space as low as the kernel size, despite of the spatial interpolation of the data. Passing through the first convolutional layer, the 3D input volumes were mapped into the 4D-feature space (as illustrated by the tesseract in Fig. 1E), where the first 3 dimensions matched the input dimensions, and the fourth dimension reflected the so-called filters that were introduced in each convolutional layer. As these feature maps passed through the network, their first three dimensions were scaled down by the factor of 2, every time they encountered an average pooling layer. Thereby, the three initial dimensions of the final feature map were scaled down to 3 × 3 × 7 (i.e., scaled down by factor of 8 in total and rounded up). On the other hand, we expanded the fourth dimension (i.e., filters) from 2 in the first convolutional layer to 3 in the following layers (i.e., 2 and 3). Therefore, the input to the flatten layer had the dimensionality of 3 × 3 × 7 × 3 (Fig. 1E).

The output of the last 3D-convolutional layer was batch normalized, flattened and passed through dropout (rate = 50%) and Rectified Linear Unit (ReLU) layers. Eventually, the output of the ReLU layer was fed into a densely connected layer with 3 neurons (corresponded to Healthy control, PD OFF MED and PD ON MED), where the “softmax” activation function was applied. Critically, unlike the common DCNN architectures, we chose to have no hidden layers in the classifier section of the network. Conventional DCNNs have multiple hidden layers after the last convolutional layer (Basha et al., 2020). However, we opted to have a minimalistic approach in our network design to increase the generalizability of our result by lowering the number of the trainable network parameters, and furthermore, to obtain feature maps in the 3D-convolutional layer that are straightforwardly related to the output layer. Therefore, following the main aim of this research, our approach was an attempt to increase the interpretability of the last convolutional layer’s output .

### 2.4 Network generalizability

Below, there is a brief summary of the steps taken to maximize the generalizability of the 3D-DCNN’s prediction, involving all levels of handling the data, designing the network architecture and finally the optimization procedure.

#### 2.4.1 Data augmentation

The primary dataset used in this study included 13,598 training samples, which were nevertheless obtained from a total of 50 individuals and therefore prone to overfitting and low generalizability. A common initial attempt to alleviate the potential overfitting problem, was applying the data augmentation which approaches the overfitting problem from its root that is the training dataset (Shorten and Khoshgoftaar, 2019). However, the inputs of our model were 3D electrophysiological activity (i.e., series of stacked topographic maps), where the maps’ orientation were important as opposed to the commonly used inputs of DCNN (e.g., a picture of a dog where the orientation of the picture does not change the entity of the object in it). Here, for example, the northern areas of the input volumes corresponded to the frontal lobe, whereas the southern sides indicated the occipital lobe, and therefore the upside-down map was not considered to be comparable with the original map. Hence, our data augmentation exclusively included random shift, zoom and elastic deformation.

**Fig. 2:**
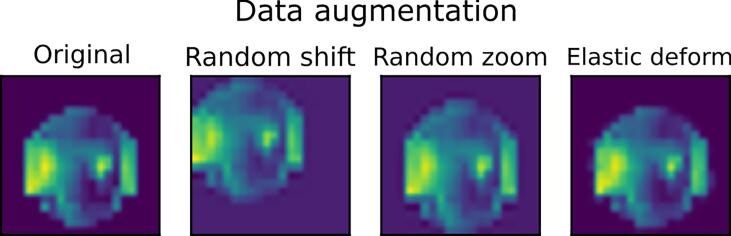
**Transformations for the data augmentation.** Three different transformations were used for the data augmentation. A representative sample is shown here as well as the output of each transformation for this given sample.

In the random shift transformation, we randomly shifted 33% of arbitrarily selected training volumes via applying a translation transformation function. The amount of translational shift was drawn from a uniform distribution, where all values between zero and 20% of the volume size were equally likely (Fig. 2). Similarly, we randomly chose another 33% of the training data and scaled them with the factors drawn from a uniform distribution in the range of 0.88 to 1.12 (Fig. 2). We repeated the random selection of another 33% of the training volumes, in order to apply an elastic deformation, where the magnitude of deformation assigned to every sample was randomly derived from a uniform distribution in the range of 0 to 1 and smoothed with a Gaussian kernel with the standard deviation of 0.08 (Fig. 2).

### 2.5 Optimizing the design of 3D-DCNN architecture

We sought to increase the generalizability of the model prediction/performance, by taking three additional steps related to designing the architecture of our 3D-DCNN, namely, the inclusion of batch normalization and the dropout layers, as well as assigning no hidden layers in the classifier section of the network. Having a batch normalization layer just after the last convolutional layer normalized the feature maps across the training batch (i.e., 25 samples per each batch) which in turn allowed the network’s training to rely on the statistical parameters and not the deterministic values of the data. Consequently this approach increased our network generalizability by improving the network's resilience against the parameter scale and reducing the probability of being trapped in a saturated regime (Ioffe and Szegedy, 2015). Furthermore, including a dropout layer with the rate of 50% in the classifier section of our 3D-DCNN helped to decrease the dependency of the network's prediction to the specific neurons at the flatten layer, via creating semi-different architectures in each training step and subsequently efficiently combining these architectures (Srivastava et al., 2014). Overall, according to the ensemble learning hypothesis, combining the predictions from multiple contributing models has been continuously found to improve performance of the machine learning methods (Srivastava et al., 2014). Finally, we decided to not include any hidden layers in the classifier part of the 3D-DCNN, to reduce the number of the trainable parameters and to further increase the generalizability of our findings.

### 2.6 Model comparison

Designing the model architecture involved choosing several hyperparameters, for example, the number of filters in each convolutional layer. In case of large datasets, hyperparameters of the network can be experimentally estimated. However, for a deeper neural network this is impractical due to the high cost of training (Han et al., 2018). The size of our sample limited the possibility to experimentally estimate the network hyperparameters and therefore we relied on a hypothesis-driven approach. Nevertheless, we systematically assessed the network's total accuracy as well as the accuracy for each dataset with regard to the accuracy of comparable architectures.

### 2.7 Employing regularization techniques for stabilizing the training

Regularization is a key step to make the network less prone to overfitting and enhance its performance for the new inputs, by modifying the cost function to shrink all the parameters (i.e., weights). We achieved this by adding the L1 and L2 regularization terms to the loss/cost function of the model in all layers of the 3D-DCNN. We chose to set the L1 and L2 regularization parameters to 0.0001 and 0.01, respectively. The L1 regularization penalizes the sum of the absolute values of the parameters (shrinks them to zero) which plays a pivotal role in the feature selection (results in dropping features associated with coefficients that go to zero). L2, on the other hand, penalizes the sum of squares of the parameters resulting in shrinking them evenly, which helps for dealing with collinear/co-dependent features. Choosing a large value for the L2 compared to the L1 was with the aim of avoiding large components in the weight matrices, which was of interest in the softmax layer where we did not want our predication to be based on a specific electrode or frequency bin. We argue that this approach increased the generalizability of our model by removing the possibility of overemphasizing a specific feature that could be exclusive to the primary dataset.

### 2.8 Data partitioning, network training and validation

Data was randomly partitioned into 75%, 12.5% and 12.5%, respectively for the training, cross-validation and testing. We trained the network with an exponentially decaying learning rate with the initial value of 1e-4. Specifically, the learning rate was exponentially lowered every 10,000 steps by:

(1) $$\mathrm{learning}\;rate=10^{-4}\times \;0.96^{\left\lfloor \mathrm{step}/10,000\right\rfloor }$$

**Fig. 3:**
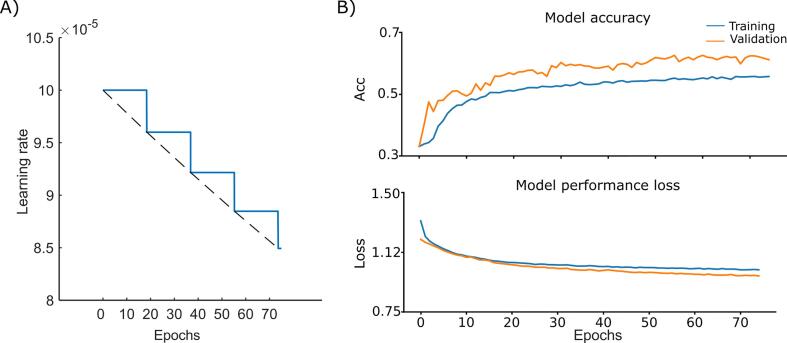
**Learning rate, model accuracy and loss. A**) The blue solid graph depicts the learning rate’s decrease as a function of the number of epochs. The dashed black line represents the base for the exponential decrement function. **B**) The accuracy and the performance loss are illustrated for the training (blue graph) and validation (orange graph) subsamples as a function of epochs. (For interpretation of the references to color in this figure legend, the reader is referred to the web version of this article.)

Where ⌊.⌋ denotes a round down operation and therefore the learning rate descended from 1e-4 in the first epoch to 8.49e-5 in the last epoch, following a staircase function (Fig. 3A). Accordingly, we achieved a finer weight adjustment during the later steps of the training.

The criteria for stopping the training of our 3D-DCNN were if the increase of accuracy and the decrease of loss were saturated or the number of training epochs reached 100. Based on these criteria we accomplished over 75 epochs of training using Adam optimizer (Kingma and Ba, 2014), Fig. 3B. It is worth noting that the dropout layer was effective only during the training but not the validation phase, and therefore, the performance loss for the validation was nearly always lower compared to the training. Moreover, given the number of training samples (i.e., 13,598) and the batch size (i.e., 25), we accomplished 40,794 iterations of training over the 75 epochs. To avoid a potential bias due to an imbalanced number of samples per class, we also included the class weights (i.e., Healthy control: 1.026, PD OFF MED: 1.007 and PD ON MED: 0.968) for estimating the accuracy and performance loss. The network parameters are summarized in Table 2.

**Table 2:** Layers and parameters of 3D-DCNN.

#	Layer	Type	Output Shape	Kernel size	Stride	Parameter #
1	Input_1	Input	(None, 20, 20, 50, 1)	–	–	0
2	Conv_1	Convolution 3D	(None, 20, 20, 50, 2)	3 × 3 × 3	1 × 1 × 1	56
3	AvgPool_1	Average pooling 3D	(None, 10, 10, 25, 2)	–	–	0
4	Conv_2	Convolution 3D	(None, 10, 10, 25, 3)	3 × 3 × 3	1 × 1 × 1	165
5	AvgPool_2	Average pooling 3D	(None, 5, 5, 13, 3)	–	–	0
6	Conv_3	Convolution 3D	(None, 5, 5, 13, 3)	3 × 3 × 3	1 × 1 × 1	246
7	AvgPool_3	Average pooling 3D	(None, 3, 3, 7, 3)	–	–	0
8	Batch_Norm	Batch normalization	(None, 3, 3, 7, 3)	–	–	6
9	Flatten	Flatten	(None, 189)	–	–	0
10	Dropout	Dropout (0.5)	(None, 189)	–	–	0
11	Re_lu	ReLU	(None, 189)	–	–	0
12	Dense	Dense	(None, 3)	–	–	570

The number of the filters for the convolutional layers is equal to the fourth dimension of the output shape for a given convolutional layer in Table 2.

### 2.9 Secondary dataset and pre-processing

We benefited from an additional open access dataset provided by Railo and colleagues on Open Science Framework (OSF, https://osf.io/pehj9/) to assess the generalizability of our network model. The data was recorded at the University of Turku, Finland. Similar to the primary dataset, it included 64-channel EEG recordings from 20 PD patients and 20 age-matched healthy controls (Railo et al., 2020). Among the PD patients, we labelled 13 individuals who voluntarily had gone on a medication break for at least 12 hours prior to the testing, as PD OFF MED. The remaining 7 PD patients were labelled as PD ON MED. Nevertheless, it is worth noting that the medication effect in the primary dataset was a within-individual variable, while in the secondary dataset, it was an inter-individual variable. The demographic information of the PD and Healthy control samples, as well as the clinical summary of the PD cohort in the secondary dataset can be found in Table 3.

**Table 3:** The demographic summary of the PD and Healthy control, as well as a summary of the PD clinical data in the secondary dataset (mean ± standard deviation).

	PD	Healthy control
N	20	20
Age	69.80 ± 7.60	67.80 ± 6.35
Gender	9 men, 11 women	8 men, 12 women
Disease duration	6.38 ± 5.25 (years)	–
LEDD^a^	663 ± 535 (mg)	–
MDS-UPDRS^b^ motor, on (n = 7)	Median: 20, range: [12, 59]	–
MDS-UPDRS motor, off (n = 13)	Median: 23, range: [12, 66]	–

a LEDD: levodopa equivalent daily dose.

b MDS-UPDRS: movement disorder society unified Parkinson’s disease rating scale.

### 2.10 Cross-dataset generalization

**Fig. 4:**
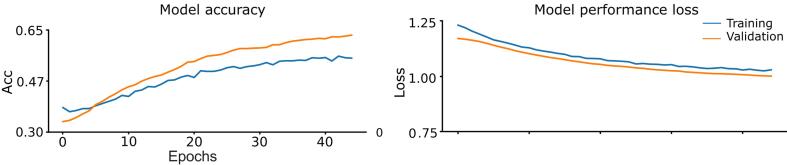
**Model accuracy and performance loss for the secondary dataset.** The accuracy and the performance loss of the 3D-DCNN showed a high transferred learning rate across the two datasets.

The deep networks are prone to overfitting especially for the datasets with a lower number of observations. Although we were cautious with this regard and took several steps (as mentioned earlier in the network generalizability section), we went further and tested performance of the network for a completely different dataset, unseen to our network model. Accordingly, we preserved the network parameters (network weights) which were estimated from the training samples of the primary dataset and sought to predict the individuals’ labels for the secondary dataset at the trial level. Nevertheless, to account for the variance across the two datasets, we applied a finetuning step only to the weights of the softmax layer. The finetuning was performed with a very low learning rate (i.e., 2e-5 as opposed to 1e-4 in the primary dataset) and a limited number of epochs (i.e., 45 as opposed to 75 in the primary dataset). The accuracy and the performance loss as a function of the training epochs are illustrated in Fig. 4. Similar to the primary dataset, we included the class weights (i.e., Healthy control: 0.651, PD OFF MED: 0.990 and PD ON MED: 2.202) to estimate an unbiased accuracy and performance loss during the training phase. Notably, the final weights were derived from the epoch exhibiting the best performance. We emphasize that only the weights of the softmax layer and not the convolutional layers were finetuned and thereby we were able to assess the generalizability of our results in the secondary dataset based on the weights from the primary dataset.

### 2.11 Network testing

There are multiple ways to measure the performance of a classifier, each can be suitable for a certain application (Krzanowski and Hand, 2009). In the current study, we used three well-known metrics to test our network performance, including the total accuracy, confusion matrix and receiver operating characteristic (ROC) curve. The total accuracy was computed within “winner-take-all” system insofar as the percentage of times that the labels were correctly assigned to the test samples. The confusion matrix was used to assess the accuracy, false positive and negative values per individual classes. To compute the confusion matrix the predicted and true labels were compared for each class. Consequently, the number of the correct and wrong labels were divided by the total number of test samples for a given cohort. Having 3 classes resulted in a three-by-three confusion matrix, where the diagonal elements are the accuracy and off diagonal elements are the false positive and negative values for a given class.

The ROC curve was the last metric we used to evaluate performance of the network. ROC is a graphical measure that shows the true positive rate on the vertical axis and the false positive rate on the horizontal axis for a given threshold. Hence, ROC is able to summarize the performance of a classifier in an effective manner where the area under the ROC curve is in fact equal to Mann-Whitney U-statistic (Krzanowski and Hand, 2009). To empirically estimate the ROC at a given threshold of “t”, we computed the true positive rate (tp) and the false positive rate (fp) as follows:

(1) $$\mathrm{tp}\;=\;p\left(S>t\left|P\right)\right),\;fp\;=\;p\left(S>t\left|N\right)\right)$$

Where S is the score estimated by the model for a given class, P is the positive true label and N is the negative true label. Therefore, functions F and G can be defined as follows:

(2) $$F\left(t\right)\;=\;1-\;tp\left(t\right),\;G\left(t\right)\;=\;1\;-\;fp\left(t\right)$$

Then from the equation 2, we were able to compute ROC at every threshold of “t” as follows:

(3) $$ROC\left(t\right)\;=\;1-G\left[F^{-1}\left(1\;-\;t\right)\right]\;\left(0⩽t⩽1\right)$$

Eventually, we computed the area under the curve from the equation 3.

### 2.12 Gradient-weighted class activation mapping (Grad-CAM)

Grad-CAM is an interpretability technique that allowed us to convert the DCNN feature maps into intuitive and understandable components (Selvaraju et al., 2019). Particularly, we utilized this property of Grad-CAM to identify components of the spectral topographic EEG maps (i.e., input volumes) that best dissociated the PD from the age-matched healthy controls, based on 1-second-long resting-state brain oscillations. Accordingly, the pre-processed spectral topographic volumes of the test samples were fed into the 3D-DCNNs and the feature maps of the output as well as the performance loss function of the last convolutional layer were extracted to compute the gradients for this layer, for each test sample. To recapitulate, the gradients of the convolutional layers (similar to the output) were indeed 4D tensors (Fig. 1E), which were scaled down by averaging over the spatial and frequency dimensions to obtain one weight per feature (i.e., last dimension). Next, these weights were used to apply a weighted average of the last convolutional layer’s output volumes, resulting in one Grad-CAM volume per a given test sample. The individual Grad-CAM volumes were then interpolated according to the original dimensions of the input volume.

### 2.13 Statistical analysis

The Grad-CAM volumes were subjected to *t*-transformation across the trials using a two-tailed independent Student *t*-test to achieve a *t*-value against zero for every voxel. Hence, analogues to the *t*-maps in the 2D data, we used the so-called *t*-volumes to identify the set of electrodes and specific frequency bands exhibiting a large effect size for dissociating each cohort (i.e., the Healthy control or PD OFF MED or PD ON MED). We identified the clusters that largely contributed to the classification of the cohorts by a conservative threshold of *t* > 20. Therefore, for a given cohort, the clusters with large *t*-values indicated spatio-oscillatory features which were critical for correct classification of the test samples. This *t*-transformation was not part of our hypothesis testing but it was required to localize the region of interest for our follow-up analysis.

We further explored these oscillatory features to assess if they were related to the clinically relevant features, using linear mixed-effects model (LMM). The power values of the pre-processed EEG data were extracted from the identified clusters (i.e., spatio-oscillatory features) and resampled to the standard normal distribution. The dependent variable of LMM was either PD duration or the off-medication UPDRS motor, or the LEDD (i.e., medication level). The fixed effect terms were age and the clusters’ normalized power. Moreover, by-participant intercept was considered as random term in the LMM (Supplementary Table S1 and S2). The parameters of the LMMs were estimated using maximum likelihood. For statistical inference, we used model comparison using the likelihood ratio test between models with and without the predictor of interest. Consequently, our inference was not affected by a potential collinearity between the predictors. The p-value for the likelihood ratio test was derived from a chi-squared test. Similarly, LMM s used for the on-medication data, where the dependent variable was the change in UPDRS motor score and the independent variables were on-medication theta power over frontoparietal, age, off-medication UPDRS and years of education. A by-participant random intercept was also included as the random term.

## 3 Results

### 3.1 Model comparison indicated a trade-off between the accuracy and the network complexity

Our approach here was to design a DCNN with a minimum number of trainable parameters. Lowering the number of parameters allows us to generate interpretable and clinically relevant topographic maps using the Grad-CAM method. Prior to assessing the individual class accuracy of the model and interpreting the output of the last convolutional layer using Grad-CAM, we examined the performance of our architecture to that of closely related designs. Critically, we were interested in the cross-dataset learning, and therefore we chose the total accuracy (the average accuracy across two datasets) as our variable of interest. We found that a mere increase of the model’s complexity did not necessarily improve the total accuracy. For example, our original design with 1,037 parameters reached a total accuracy of 59.54%, whereas a comparable design with 1,308 parameters underperformed and reached to a total accuracy of 59.49% (Table 4).

**Table 4:** Comparison of DCNN models.

#	Change	# Trainable Parameters	Accuracy Dataset 1	Accuracy Dataset 2	Total Accuracy	Complexity
1	Filter conv layer3 = 2	766	54.58	58.30	56.44	+
2	No ReLU	1,037	55.20	63.44	59.32	++
3	Original	1,037	58.46	60.63	59.54*	++
4	Filter conv layer3 = 4	1,308	61.02	57.96	59.49	+++

* Highest accuracy among the compared architectures.

### 3.2 A sub-second resting-state EEG data can identify PD and medication effect

**Fig. 5:**
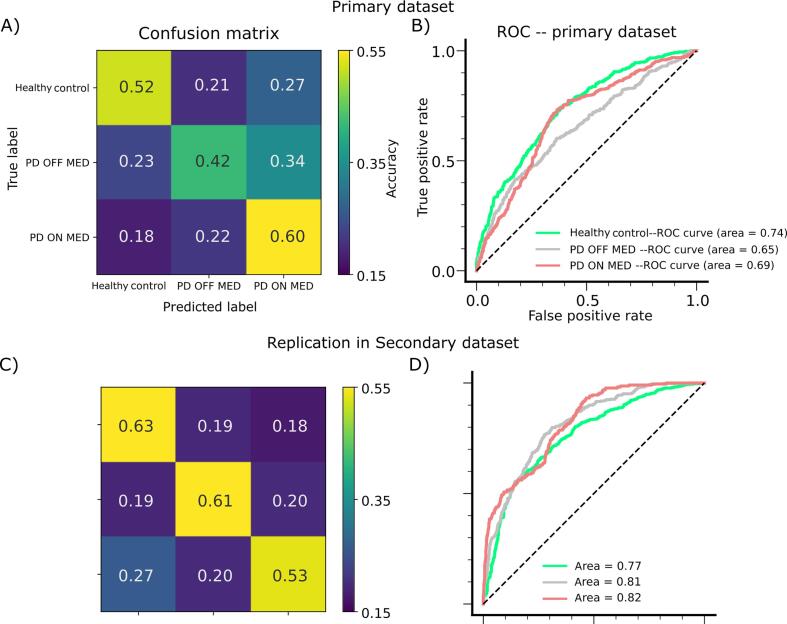
**The confusion matrix and ROC curves across two datasets. A**) The heatmap indicates the color-coded probabilities of the prediction for each class in the primary dataset. The horizontal axis represents the predicted label whereas the vertical axis denotes the true label. Consequently, the diagonal probabilities are the individual class accuracy whereas the off-diagonal values indicate the probabilities of erroneous prediction of each class. The warmer colors show higher probabilities, and the cooler colors show lower probabilities. Note that the chance level is 0.33. **B**) The graphs denote the receiver operating characteristic (ROC) curves for the three classes where the classification ability of the 3D-DCNN is illustrated as a function of discrimination threshold. Each class is color-coded and the corresponding label of each color as well as the area under the ROC curves can be found in the in right bottom corner legends. The dashed diagonal black line depicts the classification based on chance. **C**) Similar to (A), the heatmap shows the replicated class accuracies in the secondary dataset. **D**) Similar to (B), the ROC curves are illustrated for the three classes of Healthy control, PD OFF MED and PD ON MED, in the replication analysis.

Given the low signal to noise ratio (SNR) of the EEG signals at the single trial level, we did not expect to achieve a high level of accuracy. Crucially, our aim was not merely classifying the cohorts, but rather to provide some insights about the underlying dissociative information regarding the spatio-oscillatory brain activity, beyond the mass univariate analysis, using a sub-second segment of the rs-EEG data. Nevertheless, initially we assessed the accuracy for classifying our sample into the three groups (i.e., Healthy control, PD OFF MED and PD ON MED). We reached a balanced accuracy level of 58%, which is substantially above the chance level (i.e., 33%, given that there were three groups). Specifically, we observed individual class accuracies of 52%, 42% and 60%, respectively for the Healthy control, PD OFF MED, and PD ON MED groups (Fig. 5A). Subsequently, we also assessed the ROC curve for all the three classes, where the areas under the ROC curves were found to be 0.74, 0.65 and 0.69, respectively for Healthy control, PD OFF MED and PD ON MED (Fig. 5B). Next, we tested the performance of the model which was trained using the primary dataset (i.e., the same network weights), on a secondary dataset which was novel to our network model. Remarkably, we could replicate the same level of total accuracy of 61% in the second dataset using the weights derived from the primary dataset. When we assessed the accuracy separately for each class, we observed that all the three cohorts, including Healthy control, PD OFF MED and PD ON MED, maintained the same level of accuracy that was substantially above the chance level for both datasets. Particularly the class accuracy in the secondary dataset for Healthy control was 63% (c.f., primary accuracy of 52%), for the PD OFF MED was 61% (c.f., primary accuracy of 42%) and for PD ON MED was 53% (c.f., primary accuracy of 60%) (Fig. 5C). Similar to the primary dataset, we also further assessed the characteristics of the 3D-DCNN for the novel data (i.e., the secondary dataset), by evaluating the ROC. Accordingly, we obtained the areas under the ROC curves to be 0.77, 0.81 and 0.82, respectively for Healthy control, PD OFF MED and PD ON MED (Fig. 5D).

### 3.3 Grad-CAM localizes dissociative regions and oscillations in PD

To this end, all the 1,134 test samples were passed through the trained 3D-DCNN to create a Grad-CAM map per test sample. We next indicated areas in the z-scored Grad-CAM maps which were above a prior threshold (t > 20) within the test samples of all three cohorts, using a two tailed one-sample Student *t*-test. Therefore, the *t*-transformation allowed us to localize the bins in space and frequency which effectively contributed to the classification of the cohorts in both datasets.

**Fig. 6:**
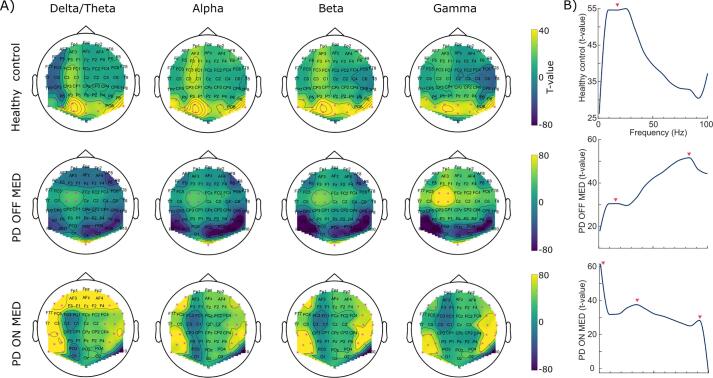
**Gradient-weighted Class Activation Mapping (Grad-CAM). A**) The plot illustrates the Grad-CAM topographic maps related to the canonical frequency bands, separately for the Healthy control, PD OFF MED and PD ON MED cohorts. The *t*-values are color-coded, so that the warmer colors indicate larger *t*-values (i.e., more impact in the dissociation of that specific group in question from the two other groups) and cooler colors represent smaller *t*-values (i.e., depicting regions that were significantly related to the other two groups rather than the specific group in question). The topographic maps of the two clusters derived from the Grad-CAM heatmaps are illustrated for (upper panel) dissociating the Healthy control group from the PD groups, (middle panel) dissociating the PD OFF MED from the two other cohorts and (lower panel) dissociating PD ON MED from Healthy control and PD OFF MED. Significant electrodes are marked with magenta asterisks. **B**) The average *t*-values of localized electrodes depicted in (A) as a function of frequency for (upper panel) Healthy control, (middle panel) PD OFF MED and (lower panel) PD ON MED. The peaks of the graphs are marked with the red triangles. (For interpretation of the references to color in this figure legend, the reader is referred to the web version of this article.)

We were critically interested to produce a heatmap highlighting the class-discriminative topographic and spectral features that allowed our 3D-DCNN to dissociate every of the three cohorts from the other classes. We operationalized this aim by using the Grad-CAM method, whereby the gradient weighted activation of the last convolutional layer (i.e., layer 3) for each test sample was computed. Next, each z-scored map was *t*-transformed across the trial dimension. We used two tailed, one sample Student *t*-test to assess the z-scored output map against zero at trial level. The assumption here is that the model has whitened the samples by removing all the variances originating from covariables of no interest, and therefore we considered each test sample as an independent observation. Specifically, the Student *t*-test was used to identify the regions at the canonical frequency bands, δ/θ (1–8 Hz), α (8–12 Hz), β (12–30 Hz) and γ (30–100 Hz), that were largely contributing to the prediction/classification of the cohorts (Fig. 6A). We found a cluster of electrodes (i.e., P9, P7, P10, PO9, PO7, PO5, PO3, PO1, POz, PO2, PO4, PO6, O1, Oz and O2) over the occipitoparietal cortex (Fig. 6A, upper panel) with a peak in the beta band (Fig. 6B, upper panel), *t* = 55, that was largely contributing to the labelling of the samples as Healthy controls. For the PD OFF MED, a more focal cluster (i.e., FC3, FC1, C3 and C1), *t* = 52, was found over left pre-motor and motor areas (Fig. 6A, middle panel) with a peak in the gamma band (Fig. 6B, middle panel). Additionally, there was another peak with a lower *t*-value in the beta band, *t* = 31. When we assessed the Grad-CAM activity for PD ON MED a widespread cluster (i.e., AF7, AF8, F7, F5, F6, F8, FC6, FT8, C4, C6, TP7, CP5, CP6, TP8, P9, P7, P5, *P*6, P8, PO7 and PO8), *t* = 62, over the frontoparietal cortex (Fig. 6A, lower panel) was found that showed the highest peak in the delta/theta band (Fig. 6B, lower panel), as well as lower peaks in the low gamma, *t* = 38, and high gamma, *t* = 32. It has to be further emphasized that the Student *t*-statistics, that were performed for this analysis, were at the trial-level. In total, we had 1,134 test samples including 369 Healthy control, 375 PD OFF MED and 390 PD ON MED samples. The peaks defined in Fig. 6B were detected using the *findpeaks* algorithm in MATLAB 2022a (The MathWorks, Natick, Massachusetts 2022)*.* Moreover, the topographic maps, illustrated in Fig. 6A, do not indicate the absolute power values, but rather the areas that their gradient weighted activity pattern were specifically favouring the prediction of any of the classes. Additionally, the *t*-transformation has been performed in one-versus-all system. The reverse direction (i.e., negative *t*-values) for a given test is the contrast of the two other superposed cohorts against the cohort in question. Hence a comparison has to be cautiously made to the previous findings on the scalp potentials.

In an ablation study, we further indicated that the specificity of the spatio-oscillatory maps decreased as we moved from deeper to shallower layers (Supplementary Fig. S1).

### 3.4 Oscillatory power is associated with PD characteristics

**Fig. 7:**
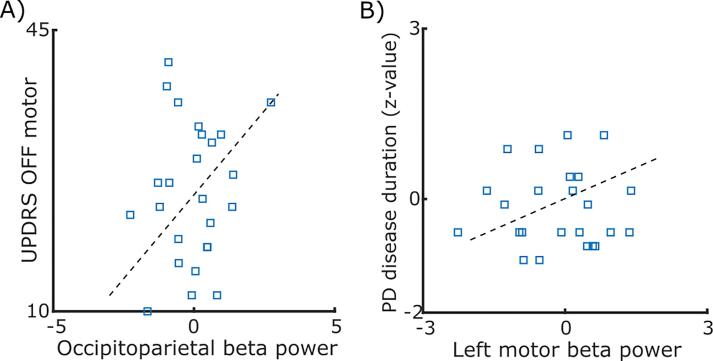
**Association of the off-medication power of the spatio-oscillatory features with the PD characteristics. A)** The blue squares represent the off-medication UPDRS motor as a function of the occipitoparietal beta power. Note that the LMM model included the by-participant random intercepts. **B**) Similarly, the blue squares represent the disease duration as a function of the beta power values over the left motor cortex. The black dashed lines show the slopes estimated by the LMM. (For interpretation of the references to color in this figure legend, the reader is referred to the web version of this article.)

To this end, using a 3D-DCNN we were able to assess the topographic maps in a multivariate framework by applying the Grad-CAM to highlight the PD- as well as medication-discriminative regions in the 3D data, spanning the space and frequency. We further asked whether the median absolute power for the distinctive clusters, either favouring the Healthy control or the PD OFF MED or PD ON MED, was associated with either the off-medication UPDRS motor score or the PD duration or the dosage of medication (i.e., LEDD) in PD cohort. Consequently, in the PD cohort, we extracted the median power from the clusters indicated in Fig. 6A, over the occipitoparietal, motor and frontoparietal cortices at the peak frequencies defined in Fig. 6B. Next, we normalized the median power values by resampling them to follow a standard normal distribution, in order to further apply the LMM. We included age and either off-medication or on-medication power values as the predictors (see the method for more details). The outcome variables were set to either the off-medication UPDRS motor, or the disease duration, or the LEDD. We used likelihood ratio test to assess whether the power oscillatory features were significantly associated with the PD characteristics and medication. We found a significant association between the off-mediation beta power value over the occipitoparietal cortex and the off-medication UPDRS motor, likelihood ratio test: *χ^2^(1) = 4.60, p* = 0.032, Fig. 7A. For more detail, please see Supplementary Table S1.

Moreover, we found that the left motor off-medication beta power was linked to the disease duration, likelihood ratio test: *χ^2^(1) = 4.24, p* = 0.039, Fig. 7B. For more detail, please see Supplementary Table S2. We did not find any effect for LEDD (*p*s > 0.149). We further indicated that the on-medication theta power over the frontoparietal area was related to the change in the UPDRS motor score (i.e., UPDRS ON – UPDRS OFF), *χ^2^(1) = 4.04, p = 0.044*. Particularly, the fixed effect of the on-medication frontoparietal theta power for prediction of the change of UPDRS score was negative, *t(20)* = −2.094, *p* = 0.049, *CI* = [−0.650, −0.001]. It is worth noting that we did not find any evidence for the association between the change of theta power (i.e., theta on-medication - theta off-medication) and the change of UPDRS score, *χ^2^(1) = 0.046, p = 0.83*.

## 4 Discussion

We proposed a multivariate approach for concurrent analysis of the oscillatory power spectrum and the topology of the scalp potentials. The primary aim of this study was to further characterize the brain electrophysiology in relation with the PD, in a data-driven and multivariate analysis framework. Accordingly, we designed a 3D-DCNN with a minimalistic architecture and conservative regularization layers with large penalized terms to identify generalizable key factors for disentangling the PD, at the expense of a lower accuracy. Subsequently, we indicated that our network outperformed models with slightly different architectures but with an approximately similar number of the trainable parameters. Moreover, our model prediction was generalizable, when tested on an independent, yet comparable dataset which was unseen to the network. Specifically, our network model which classified the cohorts in the primary dataset with the accuracy of 58%, showed a high transferred learning rate and reached even slightly better classification accuracy (i.e., 61%) in the novel dataset. Critically, using the Grad-CAM technique, we identified distinctive topographic maps and their respective predictive frequency components, that could dissociate every cohort (i.e., Healthy control, PD OFF MED and PD ON MED) from the two other groups. Particularly, we observed that the beta band activity of the occipitoparietal cortex had the highest predictive value for the dissociation of the Healthy control individuals, whereas mostly the gamma power of the left motor cortex dissociated the PD OFF MED from the two other cohorts. Finally, the frontoparietal power in theta band was the best predictive of PD ON MED. We further indicated that the off-medication beta power of occipitoparietal and left motor clusters were respectively associated with the off-medication UPDRS motor score and disease duration. Also, the on-medication theta power over frontoparietal area was negatively associated with the change of the UPDRS score.

The EEG signals are notorious for having a low SNR as well as being susceptible to the artifacts (Ball et al., 2009), and therefore they are often assessed via averaging of baseline-normalized trials, to increase the SNR, especially as for example in event related potential studies (Michel and Murray, 2012). However, it has been demonstrated that a single-trial can be imperative for the specificity in medical diagnostics and prognostic purposes, as well as following the treatment/training efficacy (Michel and Murray, 2012). Additionally, there are several different types of normalization methods and limitless strategies for the selection of the baseline interval, that may affect the results. Moreover, the intrinsic as well as preparatory spatiotemporal dynamics are considered to be irrelevant and also their strong interplay with task-evoked activity have been glossed over. Indeed, it has been known that not only there is a strong interdependency between intrinsic brain activity and task-evoked activity (Bolt et al., 2018), but also there are anticipatory adjustments in the spatiotemporal dynamics of the brain prior to a planned task (Park et al., 2012).

The influence of the reference is another critical issue in the applications of EEG, particularly considering the diversity of choices in the present EEG studies which seriously undermines the reproducibility and comparability of results. Nevertheless, regardless of the reference electrode, the pattern of the EEG topographical maps remains sustained (Geselowitz, 1998). Hence, the disproportionate stress on the waveform of the EEG regardless of the topology of the scalp potentials has been hampering the analytic power of EEG (Michel and Murray, 2012) and therefore multivariate analysis of topology of rs-EEG potentials are demanded. In the current work, we relied on a 3D-DCNN to assess the scalp topographic maps in Healthy control and PD at the single trial level. Moreover, our method allowed us to assess the EEG spectrogram using a less reductionistic and multivariate approach, enabling the concurrent analysis of spatial and frequency information. Given the noisy nature of EEG signals at the single trial level, we did not expect to achieve a high level of accuracy. Crucially, our aim was not merely classifying the cohorts, but rather to provide some insight on the underlying dissociative information regarding the spatio-oscillatory brain activity, beyond the mass univariate analysis, using a sub-second segment of rs-EEG data.

The resting-state oscillatory dynamics of PD is nearly always processed either in a continuous way (Yi et al., 2017) or using quite long segments with the duration of 80 s (Han et al., 2013) to the minimum of 2 s (Melgari et al. 2014; Neufeld et al. 1988; Oh et al. 2018; Soikkeli et al., 1991; Lee et al. 2021). Notwithstanding, a previous study with a small sample size reported that the assessment of nonlinear dynamics of 1-second-long segments of the rs-EEG can dissociate the healthy control from PD (Lainscsek et al., 2013). Yet, the 1-second-long trials in the aforementioned study were related to the baseline of a virtual grasp task while the participants were waiting for the “go” cue (Lainscsek et al., 2013). Therefore, it is not clear to what extend the anticipatory brain activity, the preparation and motivation for performing the task have affected their results and the classification accuracy (Lainscsek et al., 2013). Another study used 1D convolutional networks with >20 k parameters on 1-second-long data to classify PD from the healthy controls with high accuracy (Lee et al., 2021). We purposely epoched the EEG data in both datasets into 1-second-long trials to indicate whether the pathological oscillatory dynamics related to the PD could be captured in a sub-second time scale, however, in the two quite large datasets with no intermittent task requirements disrupting the resting-state activity. Moreover, our model has 20-fold lower number of parameters compared with the network in Lee and colleagues’ study. In the current study, we chose to have lower number of parameters due the notion that we strived to create clinically interpretable and robust topographic maps rather than achieving a high accuracy.

The fact that we were able to classify the Healthy control from the PD OFF MED based on 1-second-long trials, suggests that the PD electrophysiological signature has a sub-second time scale. Notably, our approach is different with the methods based on sliding windows where there are often considerable overlap between the consecutive windows. We complemented our network modelling by applying Grad-CAM (i.e., a class-discriminative localization technique) to interpret the deep learning model trained to classify the PD subgroups and the Healthy controls, and to identify the most distinctive electrophysiological components with the highest predictive value for each cohort. This was yielded according to the 3D Grad-CAM heatmaps localizing brain regions and respective oscillations which were critical for discriminating any group. Therefore, our multivariate approach provided us with the possibility to characterize the PD oscillatory signatures in space and frequency.

Several lines of inquiry have separately found that beta oscillations are tightly linked to the PD, although there is no consensus on the exact effects of PD on the cortical beta power (Bosboom et al., 2006, O’Keeffe et al., 2020, Pollok et al., 2012, Stoffers et al., 2007, Vardy et al., 2011, Vinding et al., 2020). Heinrichs-Graham and colleagues reported lower beta amplitude over the premotor area in the PD patients compared with the healthy controls, in both medication on and off periods (Heinrichs-Graham et al., 2014), which was largely alleviated after taking the PD medication (Heinrichs-Graham et al., 2014). Specially, the administration of l-dopa, a common PD medication, has been indicated to bilaterally increase the beta power over the motor cortex (Cao et al., 2020). Overall, there are reports of increased cortical beta power following the administration of dopaminergic medication (Heinrichs-Graham et al., 2014, Melgari et al., 2014). Based on our Grad-CAM maps, we found a cluster over the occipitoparietal with a peak in beta band where the off-medication power was associated with the off-medication UPDRS. Relatedly, a pervious computational modelling study found a high level of hazardousness for the occipitoparietal areas of the cortex, indicating that any malfunction in these regions largely affects the whole-brain function and dynamics (Kaboodvand et al., 2019). Hence, impaired oscillatory activity in the occipitoparietal cluster delineated in this study can lead to a large-scale disturbance of the brain function, which in turn can explain the motor and non-motor symptoms of PD. Furthermore, another cluster was found over the left motor cortex with a predictive value in the beta and gamma band for the PD OFF MED. Notably, an excessive phase amplitude coupling between the beta and gamma bands over the motor area has been related to the PD (Gong et al., 2021, Swann et al., 2015) which is reduced in the on-medication PDs (Miller et al., 2019, Swann et al., 2015). Moreover, the level of decrease in phase amplitude coupling has been linked to the motor improvement (Miller et al., 2019). Nevertheless, the gamma component of the reported phase amplitude coupling in the PD literature seems to be arisen from the non-linearity of the beta oscillations rather than the actual neural firing (de Hemptinne et al., 2015, de Hemptinne et al., 2013, van Wijk, 2017). In line with this notion, we observed that only the beta power, but not the gamma power, of the motor cluster was positively correlated with the disease duration.

It has been previously shown that the convolutional neural networks are able to detect medication related changes in EEG signals of the PD patients (Lee et al., 2021). In the current study, using our Grad-CAM analysis, we further demonstrated that the theta power over the frontoparietal cortex had the largest predictive value for the PD on-medication across two datasets. Moreover, using a recently developed non-invasive measure of human olfactory bulb (Iravani et al., 2020), we previously demonstrated that the odor evoked theta power recorded from the olfactory bulb, a key node in the orbitofrontal cortex, was lower in the PD patients on-mediaction compared to the healthy control (Iravani et al., 2021). Altogether, our findings support that the frontoparietal regions are largely affected by the PD medication. Crucially, we found a negative association between on-medication frontoparietal theta power and the change in UPDRS motor score. However, when we assessed the relationship between change in the theta power and change in the UPDRS motor score, we did not find any significant effect. This can be due to the notion that theta power in off-medication, as suggested by our Grad-CAM finding, is overwhelmed by the atypical motor cortex activity.

To recapitulate, the LMM analysis that we conducted to assess the relationship between the oscillatory features and the clinically relevant PD characteristics benefited from our data-driven multivariate clustering of the electrodes that had been provided by our 3D-DCNN. Specially, the patterns of scalp potentials were taken into the account, as opposed to the conventional univariate EEG analysis.

In the current study we utilized the strength of deep learning to characterize the patho-electrophysiology in PD. Nevertheless, the computational power of our analysis was limited due to having a small number of individuals in both datasets. We endeavoured to minimize the effect of small sample size by taking key steps at different phases of the study, from data augmentation, designing and training the network to replicating the results. Our achieved transferred learning indicates that our approach was able to produce generalizable results in spite of the relatively low sample size and different diagnostic criteria. Our model at its current form, is agnostic to the experiment design (i.e., between or within individual design). Nevertheless, this issue is not confounding our model given that the results were generalizable in both designs. Likewise, there is no evidence that the patient-specific features have contributed to the classification considering that using the same weights from the primary dataset, we were able to replicate same level of accuracies in the secondary dataset. Yet, future studies with larger datasets are required to not only validate our findings, but also to expand this research and localize the specific oscillatory components (i.e., scalp Grad-CAM) in different PD subtypes, for example, using representational similarity analysis.

In conclusion, we used a highly data-driven and multivariate approach, benefiting from deep learning methods, to assess the oscillatory characteristics of PD based on brief (1-second-long) periods of rs-EEG. We indicated that the PD pathological oscillatory dynamics had a sub-second time scale, considering that our network was able to classify the data above the chance level using 1-second-long rs-EEG trial. We indicated that the power of intrinsic brain activity in the beta band over the occipitoparietal, beta/gamma band over the left motor cortex, as well as the theta band over the frontoparietal cluster were respectively critical for dissociating Healthy control, PD OFF MED and PD ON MED. While the occipitoparietal beta was related to the off-medication PD motor symptoms, the off-medication motor cortex activity in the beta band was associated with the PD duration. Moreover, the on-medication frontoparietal theta power predicated the medication-related improvement of motor symptoms in PD. Our results further elucidated the patho-electrophysiology in the PD.

Code availability

The code for the training and testing of the 3D-DCNN is shared on an open access repository at [https://github.com/Behzad-Iravani/DL-on-PD-oscillaroy-topographic-pattern.git].

## CRediT authorship contribution statement

**Mehran Shabanpour:** Methodology, Software, Writing – original draft, Formal analysis, Writing – review & editing, Visualization. **Neda Kaboodvand:** Conceptualization, Methodology, Software, Data curation, Validation, Writing – review & editing, Supervision, Funding acquisition, Project administration. **Behzad Iravani:** Conceptualization, Methodology, Software, Visualization, Writing – review & editing, Supervision, Funding acquisition, Project administration.

## Declaration of Competing Interest

The authors declare that they have no known competing financial interests or personal relationships that could have appeared to influence the work reported in this paper.

## Data Availability

We used two open access datasets that are already shared on public repository. The links to the data have been provided in the manuscript.
