# Supplementary material for: Parkinson's disease is characterized by sub-second resting-state spatio-oscillatory patterns: A contribution from deep convolutional neural network
Source: Neuroimage Clin. 2022 Nov 13;36:103266. doi: 10.1016/j.nicl.2022.103266 (PMC9723309; doi:10.1016/j.nicl.2022.103266)
Supplement: Supplementary data 1 [file mmc1.docx]

**Parkinson's disease is characterized by sub-second resting state spatio-oscillatory patterns: a contribution from deep convolutional neural network**

Mehran Shabanpour, Neda Kaboodvand, Behzad Iravani

***Table S1. Linear mixed effect model for motor UPDRS off medication and oscillatory features***

| Linear mixed effects model (LMM) | Oscillatory features (suggested by Grad-CAM) | | | df | Log likelihood | Δdf | p-value |
| --- | --- | --- | --- | --- | --- | --- | --- |
|  | Frequency band | Location | Sensitive to |  |  |  |  |
| Null: UPDRS_OFF ~ 1 + Age + (1\| Participant) | ­ – | ­ – | – | 4 | -88.758 | ­ – | ­ – |
| UPDRS_OFF ~ 1 + Age + POWC1 + (1\| Participant) | Beta | Occipitoparietal | Control | 5 | -86.455 | 1 | 0.032* |
| UPDRS_OFF ~ 1 + Age + POWC2+ (1\| Participant) | Beta | Left motor | PD OFF MED | 5 | -88.555 | 1 | 0.524 |
| UPDRS_OFF ~ 1 + Age + POWC3+ (1\| Participant) | Gamma | Left motor | PD OFF MED | 5 | -88.724 | 1 | 0.793 |
| UPDRS_OFF ~ 1 + Age + POWC4+ (1\| Participant) | Delta/Theta | Frontoparietal | PD ON MED | 5 | -88.184 | 1 | 0.283 |
| UPDRS_OFF ~ 1 + Age + POWC5+ (1\| Participant) | Beta | Frontoparietal | PD ON MED | 5 | -88.718 | 1 | 0.776 |
| UPDRS_OFF ~ 1 + Age + POWC6+ (1\| Participant) | Gamma | Frontoparietal | PD ON MED | 5 | -88.758 | 1 | 0.987 |

**Table S2. Linear mixed effect model for** **disease duration and oscillatory features**

| Linear mixed effects model (LMM) | Oscillatory features (suggested by Grad-CAM) | | | df | Log likelihood | Δdf | p-value |
| --- | --- | --- | --- | --- | --- | --- | --- |
|  | Frequency band | Location | Sensitive to |  |  |  |  |
| Null: YrsSinceDiagnosis ~ 1 + Age + (1\|Participant) | ­ – | ­ – | – | 4 | -34.954 | ­ – | ­ – |
| YrsSinceDiagnosis ~ 1 + Age + POWC1 + (1\| Participant) | Beta | Occipitoparietal | Control | 5 | -33.701 | 1 | 0.11 |
| YrsSinceDiagnosis ~ 1 + Age + POWC2 + (1\| Participant) | Beta | Left motor | PD OFF MED | 5 | -32.832 | 1 | 0.039* |
| YrsSinceDiagnosis ~ 1 + Age + POWC3 + (1\| Participant) | Gamma | Left motor | PD OFF MED | 5 | -33.078 | 1 | 0.052 |
| YrsSinceDiagnosis ~ 1 + Age + POWC4 + (1\| Participant) | Delta/Theta | Frontoparietal | PD ON MED | 5 | -34.877 | 1 | 0.695 |
| YrsSinceDiagnosis ~ 1 + Age + POWC5 + (1\| Participant) | Beta | Frontoparietal | PD ON MED | 5 | -33.679 | 1 | 0.110 |
| YrsSinceDiagnosis ~ 1 + Age + POWC6 + (1\| Participant) | Gamma | Frontoparietal | PD ON MED | 5 | -33.351 | 1 | 0.073 |

**
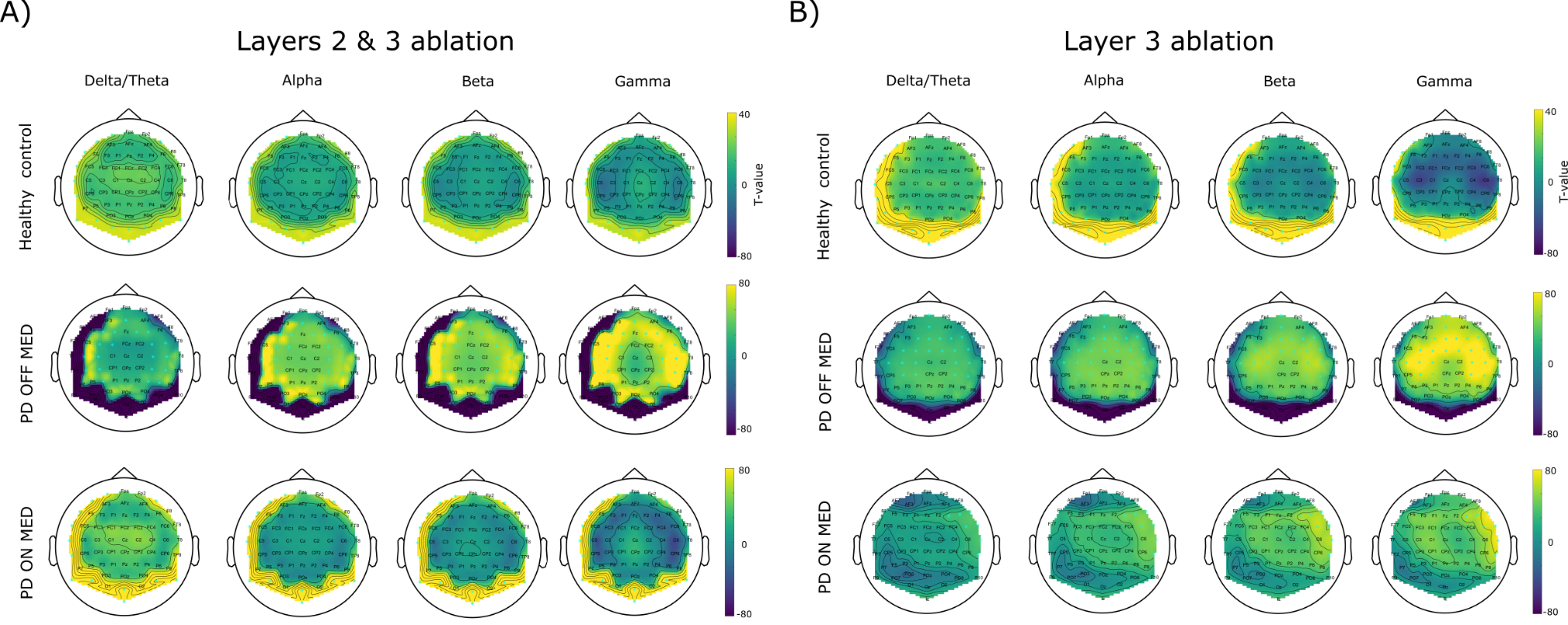
**

**Figure S1. Ablation of the deeper layers decreased the spatio-oscillatory specificity. A**) The topographical maps for ablating of the convlutional layers 2&3 and **B**) layer 3.
